# Epigenetic Changes Associated With Obesity-related Metabolic Comorbidities

**DOI:** 10.1210/jendso/bvaf129

**Published:** 2025-08-04

**Authors:** Ionel Sandovici, Tiago Morais, Miguel Constância, Mariana P Monteiro

**Affiliations:** Department of Obstetrics and Gynaecology, National Institute for Health Research Cambridge Biomedical Research Centre, Cambridge CB2 0SW, UK; Metabolic Research Laboratories and MRC Metabolic Diseases Unit, Wellcome Trust-MRC Institute of Metabolic Science, University of Cambridge, Cambridge CB2 0QQ, UK; Centre for Trophoblast Research, Department of Physiology, Development and Neuroscience, University of Cambridge, Cambridge CB2 3EG, UK; Unit for Multidisciplinary Research in Biomedicine, School of Medicine and Biomedical Sciences, University of Porto, Porto 4050-313, Portugal; ITR—Laboratory for Integrative and Translational Research in Population Health, Porto 4050-600, Portugal; Department of Obstetrics and Gynaecology, National Institute for Health Research Cambridge Biomedical Research Centre, Cambridge CB2 0SW, UK; Metabolic Research Laboratories and MRC Metabolic Diseases Unit, Wellcome Trust-MRC Institute of Metabolic Science, University of Cambridge, Cambridge CB2 0QQ, UK; Centre for Trophoblast Research, Department of Physiology, Development and Neuroscience, University of Cambridge, Cambridge CB2 3EG, UK; Unit for Multidisciplinary Research in Biomedicine, School of Medicine and Biomedical Sciences, University of Porto, Porto 4050-313, Portugal; ITR—Laboratory for Integrative and Translational Research in Population Health, Porto 4050-600, Portugal

**Keywords:** obesity, obesity comorbidities, epigenetics, DNA methylation, environmental factors

## Abstract

Obesity arises from a complex interaction of genetic, hormonal, dietary, and behavioral factors that drive chronic energy imbalance, excessive fat accumulation, systemic inflammation, and insulin resistance, thus increasing the risk of metabolic diseases. Recent evidence suggests a significant role for epigenetic mechanisms, such as changes in patterns of DNA methylation, histone modifications, and chromatin accessibility, in the aetiology, progression, and intergenerational transmission of obesity risk. In this review, we first explore the link between cellular metabolism and epigenetics in the context of an obesogenic environment and highlight the mechanisms leading to cell-type and sex-specific epigenetic changes. We then highlight recent human studies that uncovered epigenetic alterations in key metabolic organs that distinguish metabolically healthy obesity from obesity complicated with insulin resistance, metabolic syndrome, type 2 diabetes, and metabolic dysfunction-associated steatotic liver disease. Mechanistic studies performed in the mouse support an important role for epigenetic mechanisms in driving the metabolic comorbidities of obesity. Given the difficulty of accessing tissues directly implicated in metabolic homeostasis, peripheral blood epigenetic biomarkers offer insights into the pathogenesis of these metabolic comorbidities of obesity and may predict their future development. The dynamic and reversible nature of obesity-associated epigenetic changes underscores their therapeutic potential. Future research should address challenges such as tissue specificity, interactions with genetic variants, and the functional impact of epigenetic alterations. Expanding studies on intergenerational inheritance, RNA modifications, and the development of epigenetic therapies hold promise for mitigating the impact of obesity-related metabolic comorbidities and informing precision interventions in clinical practice.

Obesity is a complex, chronic disease defined as an excessive accumulation of adipose tissue that poses a risk to health [1]. The positive energy balance model proposes that obesity arises when energy intake consistently and chronically exceeds energy expenditure, leading to excessive fat storage [2, 3]. Factors that influence energy balance include genetics [4], hormone regulation [5, 6], diet composition [7], and environmental and behavioral aspects such as portion sizes [8] and sedentary lifestyles [9, 10]. In addition to the energy balance model, the carbohydrate-insulin model suggests that high-carbohydrate diets lead to hyperinsulinemia, promoting fat storage over oxidation in lean tissues [11, 12]. Obesity is associated with a wide range of pathophysiological changes in many cell types, tissues, and organs, including systemic inflammation, insulin resistance, and alterations in hormonal signaling [13-15]. These changes increase the risk of comorbidities such as type 2 diabetes (T2D), cardiovascular disease, metabolic dysfunction-associated steatotic liver disease (MASLD; formerly known as nonalcoholic fatty liver disease), some types of cancers, dementia, and other adverse pathological conditions [16]. These comorbidities increase the risk of early mortality and pose an important economic burden on those affected and society [16-18].

Among the mechanisms leading to the development and progression of obesity-related metabolic comorbidities, epigenetic changes may play a significant role. The concept of epigenetics refers to “the structural adaptation of chromosomal regions so as to register, signal or perpetuate altered activity states” caused by mechanisms other than changes in the underlying DNA sequence [19]. These structural adaptations are brought about by reversible epigenetic marks that modulate genome function, such as chemical modifications of DNA, posttranslational modifications of histones, histone variants, and alternative nucleosome positioning [20]. These epigenetic marks are laid on the chromatin by a complex family of chromatin and DNA-binding proteins with enzymatic activities, as well as noncoding RNAs [20]. Many epigenetic modifications are dynamic and are directly responsive to environmental exposures [21]. Epigenetic alterations are also known to interact with genetic factors [22] and may even be transmitted across generations [23], thus playing potentially important roles in the etiology and pathogenesis of obesity and its comorbidities.

In this mini-review, we first explore the link between obesogenic environments and epigenetic changes via alterations in cellular metabolism. These alterations often occur in a sex-dependent and cell-type-specific manner. We then review recent studies that highlight epigenetic alterations in obesity-related metabolic comorbidities in key tissues involved in the homeostasis of metabolism. Finally, we discuss emerging research areas and potential approaches to better understand the role of epigenetics in the pathogenesis and prevention of obesity-related metabolic comorbidities.

## Obesity-related Metabolic Comorbidities

Approximately 10% to 20% of all individuals with obesity are metabolically healthy, with insulin sensitivity comparable to those of healthy, normal-weight individuals [24]. Over time, approximately three-quarters of individuals with obesity develop metabolic comorbidities such as insulin resistance, metabolic syndrome (MetS), T2D, and MASLD [24].

Insulin resistance (IR), defined as a defect in insulin-mediated control of glucose metabolism in key organs such as the muscle, fat, and liver, is 1 of the earliest metabolic comorbidities of obesity [7]. IR plays a central role in the development of many other obesity-related metabolic comorbidities. IR leads to increased hepatic glucose output—driven largely by the enhanced gluconeogenesis and reduced glucose uptake into muscle cells and adipocytes—due to an impaired translocation of intracellular GLUT4-containing vesicles to the plasma membrane [7]. When the pancreas fails to supply an excess of insulin to counteract the effects of IR, blood glucose levels start to rise Consequently, impaired fasting glucose and impaired glucose tolerance appear—2 of the defining features of prediabetes and T2D [25].

Glucose is normally used as the main source of daily energy in the liver and muscle. The impaired cellular glucose uptake response to insulin induces a switch to the utilization of free fatty acids made available by the increased lipolysis in the white adipose tissue, together with the increased hepatic de novo lipogenesis [26]. The liver disposes of free fatty acids through mitochondrial β-oxidation, re-esterification into triglycerides, storage in lipid droplets, or release as very low density lipoprotein cholesterol into the systemic circulation. Over time, these processes wear out, and intrahepatic accumulation of triglycerides occurs. The excessive intrahepatic lipid deposition and inflammation in the absence of harmful alcohol intake characterize MASLD, which is observed in approximately a third of individuals with obesity [27]. MetS includes clustering of abdominal obesity, dyslipidemia, hypertension, and dysglyaemia in the form of prediabetes or T2D [28], which often coexists with MASLD [24].

## The Obesogenic Environment: Links Between Diet, Physical Activity, Hormonal Milieu, Cellular Metabolism, and Epigenetic Alterations

**Figure 1.**
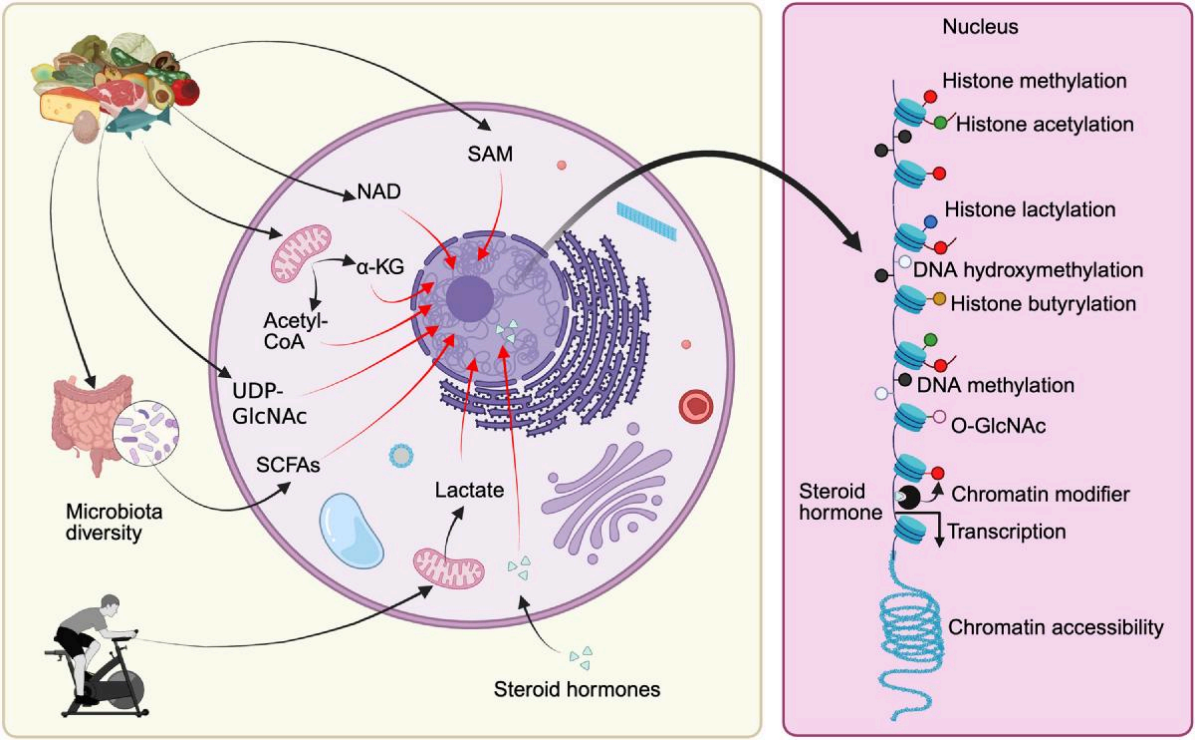
The cellular metabolic state determines the availability of key cofactors for epigenetic enzymes, thus coupling environmental inputs (diet, microbiota, exercise, hormones) to epigenetic marks and chromatin remodelling. Histone and DNA modifications depend on specific metabolites: histone acetylation on acetyl-CoA, methylation on SAM; demethylation α-KG; histone deacetylation (eg, by sirtuins) on NAD+, O-GlcNAcylation on UDP-GlcNAc, butyrylation on butyryl-CoA, and lactylation on lactate (see text for details). Lipophilic hormones (eg, estrogen, glucocorticoids) pass through cell membranes via passive diffusion. In the nucleus, steroid hormones can interact with chromatin modifiers, inducing epigenetic changes that influence transcription of target genes. Abbreviations: α-KG, α-ketoglutarate; SAM, S-adenosylmethionine.

The excessive nutrient intake, inadequate diet composition, and reduced physical activity create an obesogenic environment that disrupts cellular metabolism. In this section, we summarize how diet, physical activity, hormones, and cellular metabolism interact to induce epigenetic modifications that influence gene expression. Cellular metabolism directly affects epigenetic marks by providing essential metabolites that act as substrates or cofactors for the enzymes that write, erase, and read epigenetic modifications (Fig. 1). Changes in diet, a reduction in physical activity, or exposure to endocrine-disrupting chemicals (EDCs) can lead to alterations in cellular metabolism, promoting the development of obesity and its related comorbidities. We also highlight the sex-dependent and cell type-specific impact of the obesogenic environment on the epigenome.

### Impact of Diet

The production of acetyl-CoA, a substrate for histone acetyltransferases, is influenced by diet. Acetyl-CoA is generated through pyruvate oxidation, fatty acid catabolism, or branched-chain amino acid degradation in mitochondria [29]. Short-chain fatty acids, particularly butyrate and propionate from gut microbiota, activate the acetyltransferase p300 [30]. NAD+ is a cofactor used by sirtuins, a family of proteins that regulate chromatin structure via histone deacetylation [31]. In the mouse, obesogenic diets alter availability of acetyl-CoA, short-chain fatty acids, and NAD+ through dysregulated lipid and carbohydrate metabolism and gut dysbiosis. The altered availability of acetyl-CoA leads to aberrant histone acetylation patterns and changes in the expression of genes involved in mitochondrial function, inflammation, and insulin resistance [32-35]. Interestingly, levels of acetyl-CoA in subcutaneous adipose tissue biopsies were higher in subjects with adult-onset obesity compared with childhood-onset obesity and were related to adenosine triphosphate citrate lyase levels, demonstrating important changes in cellular metabolism with age [35].

S-adenosyl-L-methionine (SAM), derived from dietary methionine, is a key methyl donor for DNA and histone methylation by DNA methyltransferases and histone methyltransferases, respectively [36]. Although methionine intake does not directly correlate with SAM levels, dietary components such as folate, vitamins B6 and B12, choline, and alcohol influence the conversion of methionine to SAM, leading to changes in levels of DNA methylation [37]. In human individuals, plasma levels of SAM were positively correlated with fat mass and truncal adiposity [38], with the amount of fat gained in overweight patients exposed to overfeeding [39] and with the fasting insulin levels and homeostasis model assessment of insulin resistance (HOMA-IR) index in participants with at least 1 metabolic syndrome risk marker [40]. In mice, knockdown of nicotinamide N-methyltransferase, which converts SAM into its byproduct S-adenosylhomocysteine, resulted in increased SAM concentrations in the adipose tissue, increased levels of histone methylation, and resistance against diet-induced obesity [41].

O-linked β-N-acetylglucosamine (O-GlcNAc) is a posttranslational histone modification found on several histones (H2A, H2B, H3, H4) [42]. O-GlcNAcylation requires UDP-GlcNAc, the synthesis of which depends on glucose, glutamine, acetyl-CoA, adenosine triphosphate, and uridine levels [43]. Two key enzymes, O-linked N-acetyl-glucosaminyltransferase and O-linked N-acetyl β-D-glucosaminidase, regulate this modification [44]. Human studies show that O-GlcNAc levels rise in muscle biopsies with increasing blood glucose [45], while obesity is associated with lower levels in pancreatic islets [46]. In mice, O-GlcNAc's role in insulin sensitivity is further supported by the following evidence: increased resistance against diet-induced obesity upon deletion of *Ogt* in muscle [45], insulin resistance and dyslipidemia upon hepatic overexpression of *Ogt* [47], and reduced lipolysis and obesity following *Ogt* overexpression in the adipose tissue [48].

Dietary lipids, including lipotoxic compounds such as ceramides, may also influence epigenetic processes. Lipids such as triglycerides, low-density lipoprotein cholesterol, and high-density lipoprotein cholesterol affect DNA methylation at CpG sites in peripheral blood cells [49]. In vitro, palmitate-treated human skeletal muscle cells exhibited increased non-CpG methylation at the *PGC1A* locus [50]. Arachidonic acid and oleic acid, when applied to cultured monocytes, induced dose-dependent DNA hypermethylation and hypomethylation, respectively [51]. Ceramide treatment further increased DNA methylation in monocytes [52]. While emerging evidence links these processes, the underlying mechanisms remain unclear.

### Impact of Physical Activity

Physical activity, especially regular exercise, enhances mitochondrial biogenesis and oxidative phosphorylation, boosting energy metabolism and increasing the production of acetyl-CoA and NAD+, which are used by the cellular epigenetic machinery [53]. The effects of exercise on epigenetic changes vary between trained and untrained individuals and differ based on the type of exercise (endurance vs resistance training) [54]. Acute exercise can induce both DNA hypomethylation and hypermethylation in human muscle, with most changes persisting for only a few hours [55]. However, some DNA methylation changes from acute resistance exercise or training persist longer, providing a “memory” effect that supports muscle hypertrophy and future adaptations in trained individuals [56-58]. These DNA methylation changes are also influenced by the carbohydrate and fat content of the diet [59].

The transcription factor PGC-1α, which regulates mitochondrial biogenesis, is transiently activated during exercise in both humans and mice and controls exercise-induced DNA methylation changes in skeletal muscle [60]. While much research on exercise-induced epigenetic changes focuses on muscle, several studies suggest that exercise may also impact DNA methylation in other tissues, though these publications are limited due to the challenge of accessing non-muscle tissues postexercise [61, 62].

In addition to DNA methylation, endurance exercise alters histone marks such as H3K4me1 and H3K27Ac in skeletal muscle, particularly at enhancer regions of transcriptionally active genes [63]. Lactate, produced during exercise, serves as a substrate for histone lactylation, a newly recognized posttranslational modification linking metabolism to epigenetic regulation [64]. Although much of the lactate is transported to the liver for gluconeogenesis via the Cori cycle, some is used locally in the skeletal muscle for histone lactylation at active enhancers [65]. Studies in mice have shown that histone modifications play a critical role in exercise-induced muscle hypertrophy, with deletion of histone methyltransferase MLL4 and that of histone demethylase LSD1 resulting in reduced and enhanced endurance capacity during exercise, respectively [66, 67].

Physical exercise improves glucose tolerance and reverses insulin resistance in people with obesity. Skeletal muscle biopsies from patients with obesity who underwent supervised endurance training showed significant changes in DNA methylation, especially in genes regulating mitochondrial function and extracellular matrix remodeling, correlating with the magnitude of the response to the intervention [68]. DNA methylation changes have also been observed in the peripheral blood cells of children with obesity following a diet and physical activity combined [69] or physical activity intervention alone [70].

### Impact of Hormonal Milieu

Numerous hormones interact with components of the epigenetic machinery to coordinate transcription and shape chromatin structure. These effects are mediated through 2 main mechanisms. First, steroid hormone receptors often interact with epigenetic modifiers—such as writers, readers, and erasers. For instance, the glucocorticoid receptor, which plays a central role in regulating liver metabolism, interacts with several histone acetyltransferases, histone deacetylases, histone methyltransferases, and DNA-modifying enzymes (reviewed in [71]). Similarly, sex hormones, which are critical for establishing the sexual dimorphism of adipose tissue, skeletal muscle, and liver metabolism [72], can also interact with enzymes that remodel DNA methylation and histone posttranslational modifications (reviewed in [73, 74]). Second, hormones can directly regulate the expression of various epigenetic modifiers. For example, in rats, the gene encoding the histone methyltransferase EZH2 contains an estrogen response element, and treatment with estradiol increased its expression [75]. Likewise, in mice, the *Dnmt3a* gene has 2 functional thyroid hormone response elements, and administration of T3 increased its expression in the brain [76]. Epigenetic modifications can, in turn, affect the expression of key hormones or their receptors. For example, mineralocorticoid receptor expression—which helps regulate blood pressure, vascular volume, and tone through actions in the kidney, heart, and vascular smooth muscle—is increased by aldosterone and reduced by histone deacetylase inhibitors [77, 78].

Given these complex connections between the hormonal milieu and epigenetic regulation, conditions involving hormonal imbalances—as well as the therapeutic use of hormones—can induce epigenetic changes that increase the risk of obesity and related metabolic comorbidities. For instance, chronic glucocorticoid overexposure in Cushing's syndrome—which is marked by fat accumulation in the face and trunk—has been associated with altered levels of H3K4me3 and H3K27ac in visceral adipose tissue biopsies, correlated with changes in gene expression [79]. Similarly, women with polycystic ovarian syndrome—characterized by hyperandrogenism, ovulatory dysfunction, polycystic ovarian morphology, and insulin resistance—exhibited altered DNA methylation patterns in skeletal muscle biopsies, which were associated with gene expression changes [80].

Environmental exposures can also disrupt the physiological hormonal milieu, thereby contributing to an increased risk of obesity and its metabolic comorbidities. In recent decades, EDCs have emerged as significant public health concerns. EDCs are defined as exogenous chemicals—including certain pharmaceuticals—or mixtures of chemicals that interfere with any aspect of hormone action [81]. Notable EDCs include bisphenol A (BPA), phthalates, organotins (eg, tributyltin), polychlorinated biphenyls, dichlorodiphenyltrichloroethane and its breakdown product dichlorodiphenyldichloroethylene, and phytoestrogens [82]. These compounds can enter the body via ingestion, inhalation, dermal contact, or water consumption. Many EDCs act as obesogens by disrupting lipid metabolism, energy balance, appetite regulation, and adipocyte development—effects often linked to epimutations that can even be transmitted across generations. For example, female mice exposed to BPA during pregnancy developed obesity due to increased food intake, and this obese phenotype persisted for at least 6 generations through both maternal and paternal germlines, despite no further BPA exposure [83]. BPA, an estrogen-like compound, enhanced the recruitment of CTCF at 2 cis-regulatory elements of the *Fto* gene in sperm, which was associated with DNA hypomethylation. These cis-regulatory elements exhibited increased interactions with the *Irx3* and *Irx5* genes—key regulators of appetite-controlling neuronal differentiation—in sperm from obese mice. Strikingly, deletion of the CTCF site at the *Fto* gene resulted in mice that maintained normal food intake and did not become obese following ancestral BPA exposure [83]. Another example involves ancestral dichlorodiphenyltrichloroethane exposure during gestation in female rats, which led to the transgenerational epigenetic inheritance of obesity. DNA methylation epimutations were detected in sperm for up to 3 subsequent generations [84, 85].

### Sex-dependent and Cell Type-specific Epigenetic Effects of Obesogenic Exposures

Sex differences influence metabolism throughout life, contributing to the varying prevalence and clinical manifestations of cardiometabolic diseases [86]. Epigenetic mechanisms contribute to the sex-biased metabolic processes, with both X and Y chromosome-linked genes playing important roles. For instance, *KDM5C* and *KDM6A*, 2 genes that escape X chromosome inactivation in women and therefore have higher levels of activity than in men, are involved in regulating histone modifications. KDM5C induces H3K4 demethylation in preadipocytes, leading to higher adipocyte differentiation potential in females. Consequently, lowering *Kdm5c* gene dosage in XX female mice to levels that are normally present in males resulted in reduced body weight and fat content [87]. KDM6A promotes H3K27 demethylation in hypothalamic neurons that govern food intake and energy homeostasis, with higher levels of H3K27me3 found at *Ngn3*, *Pomc*, and *Npy* promoters in male neurons [88]. Similar to its homologue KDM5C, chromosome Y-linked KDM5D promotes H3K3me3/2 demethylation, though it is less active than KDM5C and may have distinct targets [89]. Autosome-linked epigenetic factors can also induce sex-biased effects. For example, deficiency of the histone variant macroH2A1.1, encoded by the *H2afy* gene located on mouse chromosome 13, results in greater obesity susceptibility and gut dysbiosis in females compared to males [90]. In mice, diet-induced changes in liver DNA methylation showed sex-by-diet interactions, highlighting the importance of sex in epigenetic responses [91]. After a high-fat diet (HFD), male but not female mice showed decreased DNA 5-hydroxymethylation in the hypothalamus, which was linked to increased body weight [92]. Many EDC exposures such as to BPA [93], polybrominated biphenyls [94], or phthalates [95] led to changes in DNA methylation patterns with sex-dimorphic patterns.

Environmental obesogenic exposures often lead to tissue-specific epigenetic changes, possibly due to a crosstalk between the environmental factors and the differences that exist in cellular metabolism. Recent advances in technology have enabled the documentation of cell type-specific epigenetic patterns, with ongoing efforts to create single-cell epigenome atlases for DNA [96, 97], histone modifications [98, 99], chromatin looping [100, 101], and chromatin accessibility [102, 103]. These patterns arise from tissue-specific differences in the expression of hundreds of genes encoding epigenetic regulators that modify DNA and histones [104]. The differential activity of epigenetic machinery can modulate the impact of cellular metabolites on the epigenome. For example, ACLY-deficient adipocytes exhibited increased acetyl-CoA levels irrespective of the depot of origin, while histone acetylation alterations were observed only in the adipocytes isolated from the subcutaneous fat depot [105]. Another key mechanism that allows environmental factors to induce tissue-specific epigenetic changes is the interaction between cellular metabolism, the epigenetic machinery, and the circadian clock [106]. For example, a ketogenic diet, which increases acetyl-CoA production and is used for weight loss, caused circadian oscillations in β-hydroxybutyrate levels in the gut but not the liver, leading to histone acetylation at PPARα-target genes in the gut [107]. Methionine metabolism also connects with the circadian rhythm [108], explaining tissue-specific changes in DNA methylation after acute sleep deprivation in humans [109]. Exercise training has also been shown to alter DNA methylation and chromatin accessibility in a tissue-specific manner [110], though the connection between these changes and the circadian clock is yet to be explored. Many EDCs induce epigenetic alterations, especially in the main target tissues of the disrupted hormones. For example, estrogen mimetics such as diethylstilbestrol induce changes in DNA methylation and H3K27ac levels, particularly in the reproductive tract [111-114]. EDCs such as pesticides, bisphenols, and phthalates are highly lipophilic, which may explain why epigenetic changes are often found in fat [115-117], liver [118-120], or brain [93, 121].

## Epigenetic Changes Associated With Obesity-related Metabolic Comorbidities in Key Metabolic Organs

**Figure 2.**
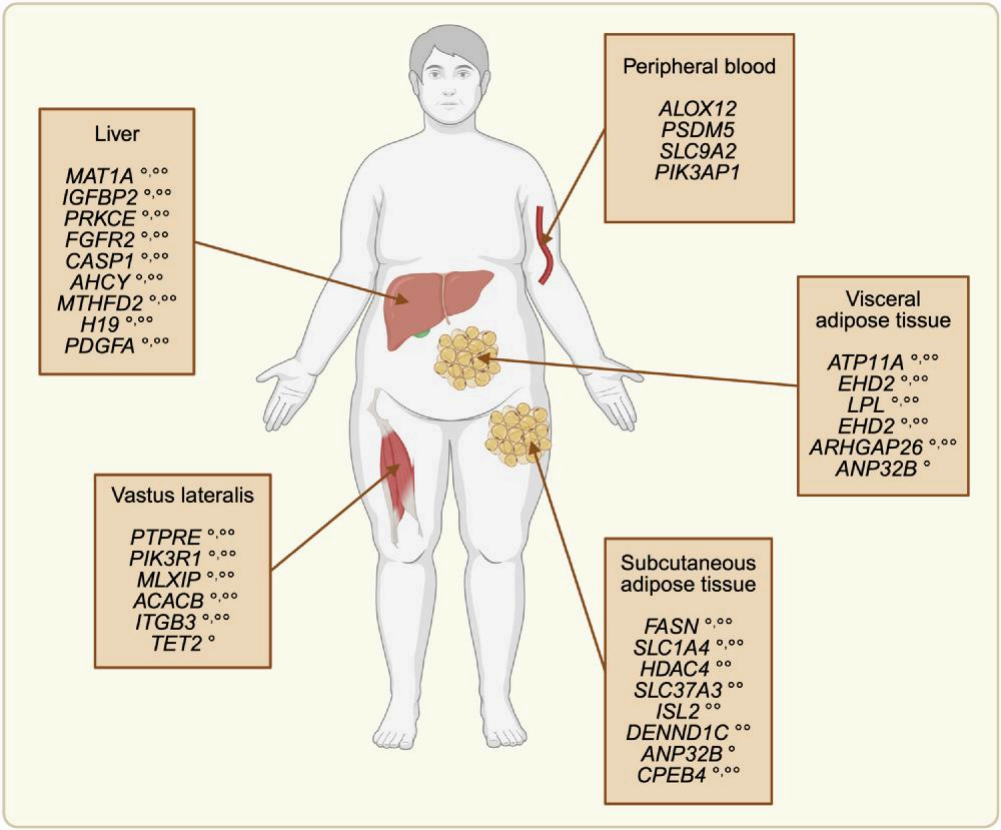
Genes with differential DNA methylation and differential gene expression that distinguish individuals with obesity-related metabolic comorbidities from those with metabolically healthy obesity. This figure illustrates examples of genes in key metabolic tissues and organs that are differentially methylated and differentially expressed in individuals with metabolically healthy obesity and individuals with obesity-related metabolic comorbidities. For some of these genes, their expression (°) or methylation levels (°°) differ between clinical subgroups or correlate with relevant metabolic parameters, such as measurements of insulin sensitivity (see also Supplemental Table S1 [122] for details and additional examples). For peripheral blood, the selected genes are those in which DNA methylation was found to be concordant with the one measured in internal organs (see also Supplemental Table S2 [122] for details).

This section highlights some of the most compelling evidence for epigenetic changes in obesity-related insulin resistance, MetS, T2D, and MASLD in key metabolic tissues and organs. These examples stem from human studies that employed unbiased, genome-scale approaches (see also Fig. 2 and Supplemental Tables S1 and S2 [122]). We complement these findings with mechanistic insights from in vivo mouse models (Table 1) and in vitro human cell studies supporting important roles played by key epigenetic modifiers in the development of obesity-related metabolic comorbidities. Our focus is on genes and loci emphasized in the original studies—those showing consistent epigenetic and transcriptional changes, functional relevance, or recurrence across cohorts—as well as representative examples from broader gene sets.

**Table 1.** Examples of in vivo mouse models demonstrating important roles played by epigenetic modifiers in obesity-related metabolic comorbidities

Gene	Epigenetic modification	Tissue/KO model	Phenotype	Mechanism	Reference
*Dnmt1*	DNA methylation	Adipocyte-specific KO	Increased fat mass, associated with increased levels of circulating insulin and serum lipid metabolites, as well as glucose intolerance	Adipocyte DNMT1 governs DNA methylation, which facilitates chromosomal looping required for *Dnm1l* expression, by preventing aberrant CTCF binding. DNM1L is a key player in mitochondrial fission, the process by which mitochondria divide	[123]
*Dnmt3a*	DNA methylation	Adipocyte-specific KO	Protection against HFD-induced insulin resistance and glucose intolerance without accompanying changes in adiposity	DNMT3A increases DNA methylation levels of *Fgf21* promoter region	[124]
*Tet2*	DNA demethylation	Adipocyte-specific KO	Protection against HFD-induced weight gain	TET2 increases hydroxymethylcytosine levels at the leptin gene promoter, thereby promoting leptin gene expression	[125]
*Ezh2*	Histone H3 lysine 27 methyltransferase	Adipocyte-specific KO	Adipocyte hypertrophy and reduced plasma VLDL-triglyceride levels	*Ezh2* deletion promotes upregulated apolipoprotein E (*Apoe*) gene expression and lipoprotein-dependent lipid uptake	[126]
*Kmt2d*	Histone H3 lysine 4 methyltransferase	Whole-body *Kmt2d*^+/−^	Resistance to HFD-induced hepatic steatosis	KMT2D/MLL4 increases H3K4me1 levels at PPRARγ2-binding sites	[127]
*Kdm3a*/*Jhdm2a*	Histone H3 lysine 9 demethylase	Whole-body KO	Obesity associated with hyperlipidemia	KDM3A decreases levels of H3K9me2 at PPAR responsive element of the *Ucp1* gene; facilitates the recruitment of PPARγ and RXRα and their coactivators	[128]
*Phf2*	Histone H3 lysine 9 demethylase	Liver-specific overexpression	Rapid onset hepatosteatosis, but also protection against liver fibrogenesis and insulin resistance in response to diet-induced obesity	PHF2 promotes the erasure of H3K9me2 on the promoter of ChREBP-regulated genes such as *Nrf2*, enhancing the oxidative stress defences	[129]
*Ehmt2*/*G9a*	Histone H3 lysine 9 methyltransferase	Liver-specific overexpression on a *db/db* genetic background	Normalization of insulin signaling and blood glucose levels in *db/db* mice, a mouse model of obesity complicated with T2D, due to a leptin receptor gene mutation	G9a promotes upregulation of *Hmga1* gene expression, a key regulator responsible for the transcription of the *Insr* gene	[130]
*Kdm2a*	Histone H3 lysine 36 demethylase	Skeletal muscle-specific KO	Protection against HFD-induced obesity and insulin resistance	*Kdm2a* skeletal muscle-specific KO leads to increased H3K36me2 levels, promoting the recruitment of MRG15 to the *Esrrg* locus, thereby reshaping skeletal muscle metabolism	[131]

Abbreviations: HFD, high-fat diet; KO, knockout; VLDL, very low density lipoprotein; T2D, type 2 diabetes.

### Adipose Tissue

Epigenetic modifications in the adipose tissue are closely linked to obesity and its associated comorbidities [132] (Fig. 2 and Supplemental Table S1 [122]). One of the largest studies on this topic examined DNA methylation and gene expression in subcutaneous adipose tissue (SAT) and visceral adipose tissue (VAT) biopsies from women with obesity and very high HOMA-IR index, as an indirect measure of insulin resistance. The study identified 29 genes in SAT and 18 genes in VAT that were differentially expressed and differentially methylated between insulin-sensitive and insulin-resistant individuals. Pathway analyses revealed enrichment in integrin cell surface interactions and insulin signaling pathways, underlining the importance of these biological processes in obesity-related insulin resistance [133]. ARHGAP26 and ANP32B emerged as common to both fat depots. ARHGAP26 seems to play a critical role in mitochondrial clearance by orchestrating localized actin remodeling and membrane dynamics, ultimately influencing metabolic flexibility under stress conditions [134]. ANP32B was identified as a histone chaperone regulating the chromatin incorporation of the histone variant macroH2A, which is known to impact heterochromatin organization, transcriptional regulation, and cellular metabolic states [135].

A similar study in SAT biopsies from men with obesity and metabolic syndrome identified 18 high-confidence candidate genes associated with diabetes and obesity traits. Several clinical traits converged at methylation loci of particular biological significance. Notably, these loci included FASN, a key enzyme involved in fatty acid biosynthesis, highlighting alterations in lipid metabolism pathways; SLC1A4, previously described as an amino acid transporter implicated in metabolic and cardiovascular disease; and CPEB4, a posttranscriptional regulator of gene expression with potential relevance for metabolic control [136]. In African American individuals with a body mass index (BMI) ranging from 18 to 42 kg/m², integrative analyses of adipose tissue DNA methylation, gene expression, and glucometabolic traits identified significant epigenetic regulatory mechanisms linking specific genes to insulin resistance and obesity. This approach highlighted 23 key genes, linked with integrin-mediated signaling, inflammation, or mitochondrial function [137]. Similarly, a separate study performed on VAT biopsies from women with obesity, with or without T2D, identified coordinated changes in DNA methylation and gene expression linked to diabetes traits, particularly fasting glucose and hemoglobin A1c levels. Of the genes identified, *ATP11A*, *LPL*, and *EHD2* showed particularly strong methylation-expression correlations. These genes are known to influence metabolic pathways, including lipid metabolism (*LPL*), cellular membrane trafficking (*EHD2*), and phospholipid translocation (*ATP11A*). Moreover, a biological pathway analysis further implicated processes such as fatty acid metabolism, aldosterone signaling, and PPARG-mediated transcriptional regulation, thereby linking epigenetic changes explicitly to T2D pathogenesis in obesity [138].

Several studies have investigated the impact of weight loss on DNA methylation in adipose tissue, particularly following gastric bypass surgery. One of these studies, performed on SAT and VAT biopsies from women who lost at least 27% of their body weight, found DNA methylation changes at thousands of CpGs, especially in SAT. Specifically, genes such as *HDAC4* (a regulator of chromatin structure and transcriptional activity), *SLC37A3* (involved in glucose metabolism and transport), and *DENND1C* (associated with vesicular trafficking and insulin signaling) exhibited methylation changes strongly correlated with fasting glucose levels [139]. In a larger cohort, the same research group identified 4 differentially methylated CpG sites (DMCs) at the *ISL2* locus linked to changes in high-density lipoprotein cholesterol as well as 2 DMCs at *PITX2* correlated with fasting glucose levels [140]. *ISL2* encodes a transcription factor recently implicated in metabolic regulation, with potential roles in lipid metabolism and oxidative phosphorylation [141]. PITX2, also a transcription factor, is involved in mitochondrial function and has been associated with lipid accumulation in metabolic tissues [142].

An interesting and relatively underexplored aspect in obesity-related metabolic comorbidities is the role of genetic variation influencing DNA methylation levels, identified through methylation quantitative trait loci (meQTL) analysis. Guénard et al conducted an epigenome-wide study in VAT biopsies from men with severe obesity discordant for MetS, identifying over 2000 significant meQTLs associated with differential DNA methylation at 174 distinct CpG sites. These CpG sites predominantly mapped to genes involved in pathways such as lipid metabolism, glucose homeostasis, and transcriptional regulation. Remarkably, 2 specific meQTLs disrupting CpG methylation within the *COL11A2* locus, which encodes a fibrillar collagen involved in extracellular matrix remodeling, were significantly associated with altered plasma fasting glucose levels [143].

Mouse studies have further elucidated the role of epigenetic modifiers in obesity-related metabolic comorbidities. Pharmacological inhibition of HDAC6, using tubastatin A, reduced food intake, fat mass, hepatic steatosis, and improved glucose homeostasis in mice with HFD-induced obesity [144]. The DNA methyltransferase DNMT3A mediates adipose insulin resistance through its downstream target gene *Fgf21,* a gene known to enhance glucose uptake and insulin sensitivity. *Dnmt3a* knockout mice were protected from diet-induced insulin resistance without changes in adiposity [124]. TET2, which catalyses DNA demethylation via oxidation of 5mC and promotes gene expression, plays a critical role in adipocyte function. A decrease in *TET2* expression is linked to hyperleptinemia in humans with obesity, and adipocyte-specific *Tet2* deficiency in mice prevents HFD-induced obesity and insulin resistance [125].

**Figure 3.**
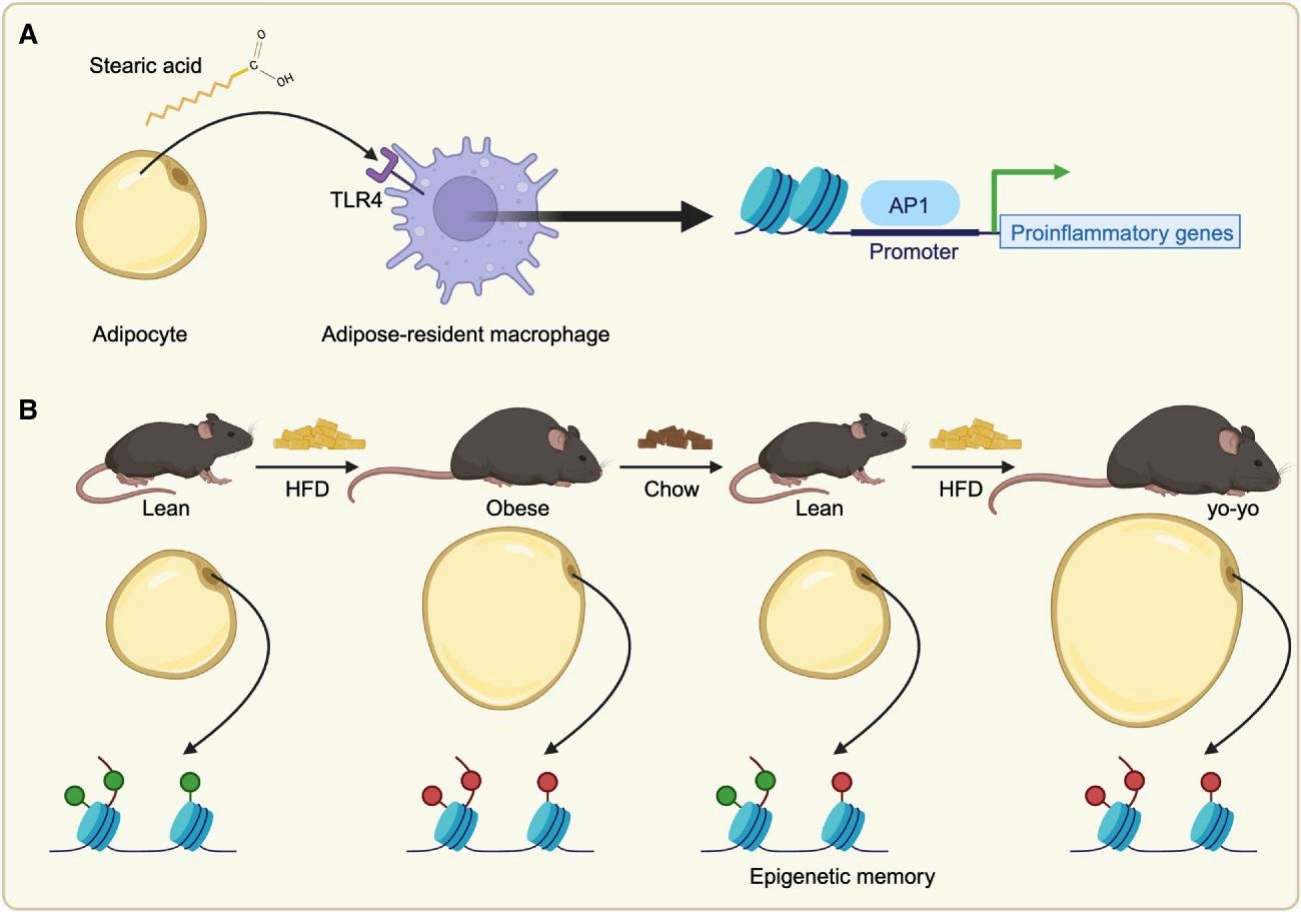
Persistent epigenetic memory after weight loss. (A) Excessive stearic acid released by adipocytes binds on the TLR4 receptor on the surface of adipose-resident macrophages leading to remodelling of chromatin accessibility at AP1 binding sites that persists upon weight loss and is responsible for enhanced transcription of proinflammatory genes. (B) A HFD induces obesity in mice, associated with altered histone modifications in adipocytes. Some of these histone alterations persist upon dietary-induced weight loss and predispose to a faster weight regain (yo-yo effect) when mice are refed HFD. Abbreviation: HFD, high-fat diet.

Further mouse studies revealed that obesity induces persistent epigenetic changes in adipose tissue that may last even upon weight loss. Excessive stearic acid produced by HFD-fed mice activates myeloid cells residing in the adipose tissue and bone marrow via their receptor TLR4, remodeling chromatin accessibility at AP1 binding sites, triggering proinflammatory cytokine production, and leading to neuroinflammation [145]. This proinflammatory phenotype persists after weight loss through stable epigenome changes (Fig. 3A) and can be passed to lean mice via bone marrow transplant [145]. Recent research also documented the “epigenetic memory” of obesity in adipocytes, which may predispose individuals to regain fat after weight loss (Fig. 3B). Male mice that were fed with HFD and then switched to a regular chow diet exhibited transcriptional obesogenic memory in adipocytes (through changes in H3K4me1, H3K27Ac and H3K4me3, and chromatin accessibility). Indirect evidence for epigenetic memory (persistent transcriptional changes) was also observed in adipocytes of individuals who experienced over 25% loss of total body weight after bariatric surgery [146]. These findings highlight the role of epigenetic changes in predisposition to obesity and related metabolic comorbidities.

### Liver

Several studies have compared DNA methylation patterns and associated transcriptome changes in the liver of individuals with obesity or obesity associated with related metabolic complications, such as MASLD and T2D (Fig. 2 and Supplemental Table S1 [122]). One of the earliest human studies analyzed the DNA methylome and transcriptome in the liver of patients with severe obesity along with various stages of MASLD and healthy controls. Integrative analysis of DNA methylation and transcriptome profiles revealed 9 genes showing both differential methylation and gene expression, including regulators of glucose and lipid metabolism and insulin/IGF-1 signaling, with most loci showing inverse methylation–expression relationships. *IGFBP2*, encoding a leptin-regulated binding protein protective against insulin resistance and hepatic fat accumulation, was downregulated and hypermethylated in nonalcoholic steatohepatitis as an advanced stage of MASLD. Additionally, *PRKCE*, encoding a protein kinase implicated in lipid-induced insulin resistance, showed altered expression and methylation [147]. Liver DNA methylation patterns can distinguish between mild and severe MASLD, with thousands of DMCs, mostly hypomethylated, linked to overexpression of genes related to tissue repair (eg, *FGFR2*, *CASP1*) and 1-carbon metabolism (eg, *AHCY*, *MAT1A*, *MTHFD2*). These genes were highlighted within pathway-level analyses, reflecting coordinated epigenetic remodeling rather than isolated gene-specific effects [148]. However, methylome and transcriptome analyses of liver biopsies from men with or without T2D (but without MASLD) revealed only subtle changes [149]. While obesity alone drove broad hypomethylation at genes regulating glycolysis and lipogenesis—particularly at activating transcription factor binding motifs—T2D-specific changes were comparatively modest. Pathway analysis revealed that genes involved in stearate biosynthesis, PI3 K/AKT signaling, and AMPK signaling were altered in both groups, suggesting that insulin resistance in obesity reflects coordinated epigenetic reprogramming of hepatic metabolic pathways. These findings align with a broader model in which obesity shifts hepatic metabolism toward a lipogenic, insulin-resistant state, regardless of overt T2D [149]. A larger study found over 250 DMCs in liver biopsies from patients with obesity with or without T2D, with most DMCs being hypomethylated in the presence of T2D. This study also identified 29 genes with both differential DNA methylation and gene expression, including the imprinted gene *H19,* a maternally expressed noncoding gene involved in growth control and hepatic lipid metabolism [150]. Another study explored the correlation between saturated fatty acids and DNA methylation in liver biopsies from individuals with obesity. Thousands of CpGs showed methylation patterns correlated with total liver saturated fatty acid content, with specific DMCs associated with MASLD, steatohepatitis, and hyperglycaemia [151].

Experimental studies have explored the role of DNA methylation in insulin resistance and obesity-related T2D. One study identified a hypomethylated DMC at the *PDGFA* locus (encoding platelet-derived growth factor α) that increases PDGF-AA secretion from the liver of patients with obesity. PDGF-AA amplifies its own expression and suppresses key components of the insulin signaling pathway, including *INSR* and *IRS1*, leading to reduced AKT phosphorylation. This cascade contributes to hyperinsulinemia, insulin resistance, and an elevated risk of steatohepatitis. It is noteworthy that these effects were reversible with PDGF-AA-blocking antibodies [152].

Additional mechanistic insights were provided by studies performed in mice. For example, dietary methyl-donor supplementation in obese mice prevented the progression of MASLD to steatohepatitis but did not revert it [153]. A study combining HFD exposure with genetically diverse mouse strains (C57BL/6J, A/J, and NOD) assessed the impact of genetic variation on epigenetic and transcriptomic responses to the environment. The researchers found that altered cholesterol biosynthesis in the liver, mediated by strain-specific regulatory networks, was linked to divergent changes in gene expression and DNA methylation. In C57BL/6J mice, the farnesoid X receptor pathway, targeted by the drug GW4064, reversed HFD-induced obesity, along with partial reversion of diet-induced transcriptome and DNA methylome changes in the liver [154].

While genome-wide epigenetic analyses of human liver focus on DNA methylation, mouse studies suggest additional mechanisms at play. For example, the histone H3 lysine 4 methyltransferase MLL4/KMT2D regulates overnutrition-induced murine steatosis by adding H3K4me1 at PPARγ2 binding sites, a key regulator of hepatic steatosis [127]. Another study highlighted the histone demethylase PHF2, which removes H3K9me2 marks from promoters of ChREBP-regulated genes. PHF2 acts as a molecular checkpoint to prevent MASLD progression during diet-induced obesity in mice [129].

### Skeletal Muscle

The muscle is the main site for glucose uptake in the body and plays a critical role in glucose homeostasis. Several studies have looked at the effects of bariatric surgery on the plasticity of DNA methylation in the vastus lateralis skeletal muscle of individuals with obesity with or without metabolic comorbidities (Fig. 2 and Supplemental Table S1 [122]). One such study performed in muscle biopsies of individuals with obesity collected before and after sleeve gastrectomy or gastric bypass surgery assessed the temporal dynamics in insulin sensitivity, transcriptome, and DNA methylome changes following surgery [155]. The most immediate impact of the rapid weight loss postsurgery was observed at the transcriptome level (as early as 2 weeks). In contrast, muscle insulin sensitivity became comparable to normal-weight controls only at 52 weeks, the slow improvement being explained by persistent elevations of intramyocellular lipid intermediates due to adipose tissue lipolysis. DNA methylation changes were very sporadic at 2 weeks, and their frequency and correlation with gene expression changes became more apparent at 52 weeks, when 2956 DMCs were identified in 921 differentially expressed genes. Of these, 4 genes—*PTPRE*, *PIK3R1*, *MLXIP*, and *ACACB*—were specifically highlighted by the authors due to their known regulatory roles in insulin signaling and energy metabolism. PTPRE is a negative regulator of insulin receptor signaling, PIK3R1 is a core component of the PI3K–Akt pathway, MLXIP governs glycolytic gene transcription, and ACACB controls mitochondrial fatty acid oxidation [155]. Another study investigated the reversibility of DNA methylation changes at 3 months after weight loss induced by gastric bypass surgery in women with obesity and insulin resistance [156]. When comparing post- vs presurgery muscle biopsies, the authors found 117 DMCs. These DMCs included the promoter of *ITGB3*, highlighted in the study as a novel gene that may contribute to the metabolic improvements observed after weight loss induced by surgery [156]. While earlier studies had implicated *ITGB3* in skeletal muscle differentiation and regeneration, more recent conditional knockout models showed no significant impairment in muscle regeneration when *Itgb3* was specifically inactivated in muscle stem cells [157]. These apparent conflicting observations suggest that any functional role in myogenesis may be context-dependent or compensated by other integrin-encoding genes such as *Itgb1* [157]. Recently, the same team investigated the plasticity of insulin sensitivity, gene expression, and DNA methylation in the skeletal muscle of individuals with obesity with and without T2D undergoing bariatric surgery. Specifically, they compared muscle biopsies at the time of surgery and 52 weeks after [158]. The authors observed that different subsets of genes were affected at mRNA level in the 2 groups of patients. Genes related to insulin, PPAR signaling, and oxidative phosphorylation pathways were altered in individuals with obesity without T2D, while genes related to ribosome and spliceosome were changed in those with T2D. DNA methylation was less affected in individuals with obesity and T2D, with about 3 times fewer DMCs. This apparent resistance was inferred to be related to reduced expression levels of DNA demethylase TET2 and higher global levels of DNA hydroxymethylation postsurgery in patients with T2D [158]. Regression analyses highlighted distinct sets of genes associated with metabolic improvement after surgery, most of which were different between individuals with obesity alone and those with obesity and T2D, reflecting a divergence in epigenetic remodeling and mitochondrial activity [158]. These findings suggest that epigenetic inflexibility in T2D may hinder skeletal muscle adaptation and limit metabolic recovery postsurgery.

### Pancreatic Islets

Obesity is 1 of the main risk factors for T2D, and several studies have investigated DNA methylation changes in pancreatic islets from donors with and without diabetes (reviewed in [159]). However, to our knowledge, there is no study that specifically compares islets from individuals with and without T2D among those with obesity. In the absence of such studies, most insights come from in vitro or animal studies. Chronic high levels of free fatty acids in obesity negatively affect β-cell function. One study exposed human pancreatic islets to palmitate for 48 hours and found impaired glucose-stimulated insulin secretion, with over 1800 differentially expressed genes and more than 4600 DMCs, mostly hypermethylated. Interestingly, 2 genes linked to T2D (*TCF7L2* and *GLIS3*) were among the genes affected at both the transcriptional and epigenetic level. TCF7L2, a transcription factor in the Wnt signaling pathway, regulates insulin production and β-cell proliferation, while GLIS3 is essential for pancreatic development and insulin gene expression. Pathway enrichment analyses highlighted disruptions in glycolysis/gluconeogenesis, fatty acid metabolism, pyruvate metabolism, and the insulin signaling pathway—pointing to impaired energy handling and insulin regulation under lipotoxic conditions [160].

In mice, long-term HFD feeding led to obesity and mild glucose intolerance, causing changes in the expression and H3K27ac levels of hundreds of genes in pancreatic islets. Many of these genes were involved in fatty acid signaling, further supported by increased H3K27ac and mRNA levels of β-oxidation-related genes in palmitate-treated INS-1 cells [161]. Another study profiled single-nuclei RNA and enhancer marks (H3K4me1 or H3K27ac) in islets from lean and obese mice. The analysis revealed β-cell heterogeneity of enhancer states in response to metabolic stress, including a subset of H3K4me1^+^/H3K27ac^−^ primed enhancers that were prevalent in lean mice and largely absent in obese mice after metabolic stress. In lean mice, these enhancers were occupied by the FoxA2 transcription factor, which plays an important role in β-cell function [162].

## Epigenetic Biomarkers of Obesity-related Metabolic Comorbidities

Due to the difficulty of directly accessing tissues regulating metabolic homeostasis, many epigenetic studies focus on easily accessible biological samples, particularly blood. One major advantage of epigenetic studies in blood is the ability to design epigenome-wide association studies (EWAS), following stringent criteria similar to those used in genome-wide association studies [163, 164]. DNA methylation is a widely used and stable epigenetic marker, making it ideal for EWAS. This section highlights key studies that explored epigenetic biomarkers of obesity-related metabolic comorbidities through DNA methylation in blood and other tissues (Fig. 2 and Supplemental Table S2 [122]).

The largest EWAS on BMI and adiposity outcomes analyzed DNA methylation in over 10 000 individuals, identifying 187 loci with significant methylation changes (*P* < 1 × 10^−7^), mostly resulting from adiposity rather than causing it. Many of these changes were in cis-regulatory regions that operate across tissues [165]. Strikingly, 62 of the loci were predictive of future T2D, with *ABCG1*, involved in insulin secretion and β-cell function, showing the strongest prediction power, remaining significant even after adjusting for adiposity and glycemic measures [165]. A meta-analysis of 6 EWAS on BMI identified 1265 significant CpG sites, with 397 explaining 32% of BMI variance. Individuals whose methylome-predicted BMI was higher than their actual BMI had worse metabolic profiles, including higher glucose and triglycerides, while those with a BMI lower than predicted had lower glucose and better lipid profiles [166].

While most DNA methylation changes in obesity-related metabolic comorbidities are tissue-specific, some loci are shared between circulating cells in blood and resident cells in tissues. For instance, a study performed on liver, VAT, and SAT biopsies, as well as peripheral blood collected from patients with obesity undergoing bariatric surgery with or without T2D, revealed correlated DMCs at *ALOX12* (blood and liver), *PSDM5* (blood and SAT), and *SLC9A2* (blood and VAT), though these did not show expression changes. *ALOX12*, which encodes an enzyme involved in inflammatory lipid signaling, has also been linked to insulin resistance in visceral adipose tissue. *PSMD5* and *SLC9A2* are involved in proteasome function and ion exchange, respectively, but their role in obesity remains to be clarified [167]. Another study in individuals with severe obesity undergoing bariatric surgery found over 1500 DMCs in the peripheral blood, overlapping with changes in SAT and VAT. This overlap represented about 3.6% of all CpGs measured, with some DMCs correlating with gene expression changes. Significantly, *PIK3AP1*, encoding an adaptor in the PI3 K signaling pathway, differentially expressed in both fat depots, correlated strongly with obesity-related traits such as HOMA-IR, fasting insulin, glucose, and triglyceride levels [168].

## Concluding Remarks and Future Directions

This mini-review highlights the emerging evidence linking environmentally induced epigenetic changes to obesity-related metabolic comorbidities. Epigenetic studies in humans are complex due to several factors. First, the epigenome is cell-type specific, requiring the study of relevant and often hard-to-access tissues. Additionally, tissue heterogeneity can mask cell-type-specific changes, despite the use of bioinformatics tools for cell-type deconvolution [169], necessitating innovative methods such as single-cell epigenomics. Second, the epigenome is dynamic, influenced by age, environment, diet, stress, and disease. Longitudinal studies are needed, though they are technically and logistically challenging. Databases such as the EWAS Catalog [170] will help accelerate understanding of the roles of epigenetic alterations in metabolic disease. Third, the functional consequences of the vast number of identified epigenetic modifications are very poorly understood. For example, linking DNA methylation changes to gene expression and predicting phenotypic outcomes is difficult, requiring new statistical and machine learning approaches [171]. The strongest evidence for a causal role of epigenetics in obesity-related comorbidities comes from mouse knockout models targeting epigenetic writers and erasers (see Table 1). Future epigenome editing of obesity-related genes may offer further causal insights. Fourth, genetic diversity among individuals influences the epigenome and complicates the association of specific epigenetic changes with diseases or traits [172].

Several emerging research areas could expand our understanding of the environmental impact on diseases and their clinical relevance. One such area is the epitranscriptome, which refers to RNA modifications (eg, methylation, acetylation, deamination, and oxidation) that regulate RNA metabolism, stability, and their interactions with other molecules like DNA and proteins [173]. Recent findings suggest a crosstalk between the epitranscriptome and epigenome, such as an influence of RNA methylation on chromatin architecture [174]. *FTO*, a gene associated with obesity, encodes an mRNA demethylating enzyme that removes the m^6^Am (N6,2′-O-dimethyladenosine) cap modification, which controls mRNA stability [175]. HFD-induced obesity in mice leads to increased FTO levels and loss of m^6^Am at specific targets such as *Fabp2* and *Fabp5* mRNAs [176]. Additionally, individuals carrying specific *FTO* risk alleles (eg, rs9939609) show DNA methylation changes at loci associated with obesity [177], though a direct link between m^6^Am levels and DNA methylation in these individuals has not been explored yet.

Another promising area of research is the intergenerational epigenetic inheritance of metabolic diseases. Human studies suggest associations between maternal traits (eg, BMI) and epigenetic changes in offspring (reviewed in [178, 179]), with studies in mice showing that maternal obesity induces epigenetic changes in both sperm [180] and oocytes [181]. These changes involve germline-specific mechanisms, such as alterations of mitochondrial transfer RNAs in sperm and abnormal DNA methylation patterns in oocytes [181]. These mechanistic differences may explain the observed sexual dimorphism in the inheritance of metabolic traits, an area requiring further investigation [182].

The reversible nature of epigenetic modifications has led to the development of epigenetic drugs (epi-drugs) targeting enzymes that add or erase epigenetic marks. The use of epi-drugs in cancer treatment has demonstrated their potential to alleviate disease [183]. Candidate epi-drugs for treating obesity-related metabolic comorbidities include inhibitors of DNA methyltransferases, compounds that target specific histone deacetylases and histone acetyltransferases, as well as sirtuin-activating compounds. Only a small number of such molecules are undergoing preclinical or clinical trials [184, 185]. However, further research is needed before epigenetic therapies can be applied to people living with obesity and its comorbidities.

In conclusion, recent studies provide compelling evidence for a mechanistic link between environmental factors, epigenetic changes, and obesity-related metabolic comorbidities. Future research should focus on characterizing epigenetic modifications beyond DNA methylation, understanding their impact on gene expression, and translating these findings into clinical practice to benefit patients.

## Data Availability

Data sharing is not applicable to this article as no datasets were generated or analyzed during the current study.
